# Impact of COVID-19 pandemic on inequalities in mortality: an analysis in Piedmont and Emilia-Romagna: Elena Strippoli

**DOI:** 10.1093/eurpub/ckac130.028

**Published:** 2022-10-25

**Authors:** E Strippoli, N Zengarini, C Di Girolamo, L Bartolini, C Aversa, G Costa

**Affiliations:** 1 Epidemiology Unit, ASL TO3 Piedmont Region, Grugliasco, Turin, Italy; 2 Health and Social Care Agency, Emilia-Romagna Region, Bologna, Italy; 3 Epidemiology Unit, Azienda USL-IRCCS di Reggio Emilia, Reggio Emilia, Italy

## Abstract

**Background:**

Italy was heavily hit by the COVID-19 pandemic. According to official statistics, during 2020 there were more than 75,000 excess deaths compared to the average expected mortality in 2015-2019. General mortality (GM) is a good measure of both the direct and indirect effects of the pandemic because it's exempt from potential bias due to misclassification of events. Evidence shows a greater burden of disease and mortality attributable to COVID-19 among disadvantaged populations, with the risk of an exacerbation of existing health inequalities. We aim to analyse the trend of social inequalities in mortality during the first pandemic year in two Italian regions (Piedmont and Emilia-Romagna) using data from Administrative Population Registries (APR) and statistical databases.

**Methods:**

Data on deaths occurred between Jan 2015 and Jan 2021 in subjects ≥65, stratified by educational level, were obtained from Regional APR and the Census. Using a time series approach, we computed Standardized Mortality Rates (SMR), Relative Index of Inequalities (RII) and Slope Index of Inequalities (SII), adjusted by age, gender, month and region. SMR, RII and SII from March 2020 were forecasted using Holt-Winters method and compared to the observed values in the same period.

**Results:**

SMRs were higher than expected during the two 2020 epidemic waves (Mar-Apr, Oct-Dec) in both regions. RII didn't increase significantly. Absolute inequalities instead rose in Piedmont during both pandemic waves, mostly among women, and in Emilia-Romagna in March among men.

**Conclusions:**

The impact of the pandemic on inequalities in GM has been at least of the same size of the impact of other mechanisms of unequal mortality. APR coupled with sociodemographic data are a quick and reliable source for assessing the unequal impact of the COVID-19 pandemic on health. Further research is needed to explore mechanisms underlying these effects e.g. inequalities in cause-specific mortality and access to health services.

**Key messages:**

- The unequal impact of the pandemic on mortality was confirmed.
- Administrative data linked with Census and health data are efficient and reliable sources for a timely monitoring of health inequalities.

